# Synthesis of Magnetic Microspheres with Sodium Alginate and Activated Carbon for Removal of Methylene Blue

**DOI:** 10.3390/ma10010084

**Published:** 2017-01-20

**Authors:** Chaodao Li, Jianjiang Lu, Shanman Li, Yanbin Tong, Bangce Ye

**Affiliations:** 1 School of Chemistry and Chemical Engineering, Shihezi University, Shihezi 832003, China; lichaodao_0412@163.com (C.L.); Lishanman8674@sina.com (S.L.); tongyanbin@sina.com (Y.T.); bcye@ecust.edu.cn (B.Y.); 2 Key Laboratory for Green Processing of Chemical Engineering of Xinjiang Bingtuan, Shihezi 832003, China

**Keywords:** magnetic microsphere, methylene blue, adsorption properties

## Abstract

Based on the adsorption performance of composite microspheres with activated carbon (AC) and sodium alginate (SA), as well as the magnetic property of Fe_3_O_4_, we designed and explored an efficient strategy to prepare a unique, multifunctional Fe_3_O_4_/AC/SA composite absorbent (MSA-AC) that extracted dye from aqueous solution. The composite exhibited the following advantages: rapid and simple to prepare, environmentally friendly process, low-cost, recyclability, and multi-functionality. The physicochemical properties of the prepared magnetic microspheres were measured, and methylene blue (MB) was selected to investigate the performance of the magnetic absorbent. The results showed a maximum adsorption capacity of 222.3 mg/g for MB. Adsorption studies revealed that the data of adsorption isotherms and kinetics fit the pseudo-second-order kinetic model and Langmuir isotherm model.

## 1. Introduction

Dyes are indispensable chemical compounds in textiles, paper-making, cosmetics and other industries, and their use leads to large quantities of wastewater [1]. Due to the number of organic contaminants, dye wastewater is toxic, carcinogenic, and causes serious environmental problems. Furthermore, dye wastewater has been linked to genetic mutation in human beings [2,3,4] because the organic contaminants can accumulate in living tissues [1,5]. The development of a method to separate and break down the individual organic constituents in order to reuse dye wastewater is not only of great importance for environmental safety but also as a sustainable development strategy [5,6].

Undoubtedly, the removal of dye from waste effluents is an environmental concern, and various dye wastewater treatment technologies have been investigated, such as chemical precipitation, adsorption [2,7,8], membrane filtration [9,10], biological treatment [11] and photocatalytic degradation [4,12]. However, most of these methods are not widely utilized because of efficiency, environmental impact and cost. Adsorption is one of the most effective and feasible methods for wastewater treatment due to its relatively low cost, ease of operation, fewer harmful secondary products [13] and toxic pollutants. Activated carbon (AC) is one of the most extensively investigated materials for the removal of various pollutants problems because of its high surface area, porous texture to accommodate large organic molecules, non-toxicity, chemical inertness, and high capability for adsorbing a broad range of contaminants [14,15,16]. Numerous studies have been conducted in an effort to develop high surface area AC from a myriad of inexpensive precursors, such as sugarcane, bamboo, straw, fruit peel, and excess sludge [1,13,17,18]. However, the optimization of the activation reaction for the adsorption of dyes is restricted because of aggregate formation and regeneration cost.

Sodium alginate (SA) is an interesting natural polymer that is abundant, biodegradable, hydrophilic, low-cost, and easily processed into various shapes [19]. SA is widely used as a bioaffinity carrier material, and due to the abundant and easily modified carboxyl and hydroxyl groups on the backbone of the molecule, it was investigated to enhance the adsorption preference of dyes [20,21]. Additionally, SA is sensitive to temperature and pH [22]. Compared with traditional hydrogels, direct preparation of SA/AC microsphere hydrogels is simpler, produces more uniform particles, and reduces AC aggregation [23]. Moreover, for dye wastewater treatment, AC and SA have attracted much attention due to their unique pore structures and extremely large surface areas, properties that allow for the effective adsorption of dyes.

While SA/AC microspheres have a large reactive surface area and high adsorption capability, their recovery and reusability from aqueous solution are crucial factors for further applications. Fe_3_O_4_, as a magnetic material, has gained much attention as a means to provide fast and efficient adsorption [5,24,25]. Yang et al. [26] prepared Fe_3_O_4_/histidine composite nanoclusters, which exhibited remarkably efficient adsorption of proteins. Wan et al. [27] successfully fabricated a hydrophilic enrichment composite that demonstrated robustness and feasibility for profiling glycoproteomics in real biological samples. With the increasing number of environmental problems, a new, facile, and environmentally friendly method of synthetic polymerization is urgently needed.

To our knowledge, there has been no report of the application of Fe_3_O_4_/AC/SA particles for adsorbing dyes. In this study, we prepared Fe_3_O_4_/SA/AC microspheres and extracted dyes from aqueous solution via a simple two-step route: synthesis of Fe_3_O_4_ by coprecipitation and synthesis of advanced composite materials incorporated with AC and SA, as well as Fe_3_O_4_. Methylene blue (MB) was selected as a target dye to investigate the performance of the magnetic microspheres because of its toxicity, mutagenicity and carcinogenic potential.

## 2. Materials and Methods

### 2.1. Materials

All chemicals and reagents were analytical grade unless otherwise stated. Sodium alginate (SA, 50%), activated carbon (AC, Charcoal active powder), granular calcium chloride (CaCl_2_, ≥97.8%) and absolute ethanol (≥99.8%) were obtained from Tianjin Fuchen Fine Chemical Industry Research Institute (Tianjin, China). Iron (II) chloride tetrahydrate (FeCl_2_·4H_2_O) and iron (III) chloride hexahydrate (FeCl_3_·6H_2_O) were obtained from J&K Scientific Ltd. (Beijing, China). Ethylenediamine (EA) was purchased from Aladdin Reagent Inc. (Shanghai, China). Methylene blue (MB), a biological stain, was purchased from Tianjin New Fine Chemicals Development Center (Tianjin, China).

#### Materials Characterization

Powder X-ray diffraction (XRD, Shimadzu XRD-7000, SHIMADZU LIMITED Hitachi High Technologies, Tokyo, Japan) analyses were performed with Cu-Kα radiation (λ = 1.5418 Å, 40 kV, 30 mA), and patterns were recorded in the range of 2*θ* = 10°–80° with a step size of 0.02°. Scanning electron microscopy (SEM) measurements were carried out on a Hitachi S-3400N detector (Hitachi High Technologies, Tokyo, Japan). Fourier transform infrared spectroscopy (FT-IR, Nicolet 380, Nanjing Lear Instrument Equipment Co., Nanjing, China) spectra were recorded by using the KBr pellet method. Magnetic properties of the samples were tested on a vibrating sample magnetometer (VSM, MPMS3 SQUID, Quantum Design, San Diego, CA, USA). Brunauer–Emmett–Teller (BET, Micromeritics asap2460, Micromeritics Instrument Corp, Atlanta, GA, USA) specific surface area of the materials was determined from nitrogen gas adsorption–desorption at the boiling point of liquid nitrogen (−196 °C).

### 2.2. Synthesis

#### 2.2.1. Synthesis of the Fe_3_O_4_

The superparamagnetic nanoparticles (Fe_3_O_4_) were obtained by the aqueous co-precipitation method with minor modifications [27,28]. In a three-necked flask, 1.72 g FeCl_2_·4H_2_O and 4.72 g FeCl_3_·6H_2_O were dissolved in 80 mL deionized water. Under a nitrogen atmosphere, the mixture was vigorously stirred (1000 rpm) at 80 °C until the salts were completely dissolved. Then, 20 mL of ammonium hydroxide (25% in water) was added dropwise to the reaction mixture with vigorous stirring. A change in color from an orange-red solution to a black suspension signified the formation and precipitation of magnetic particles. After 30 min, the reaction was stopped and the suspension was cooled to room temperature. The final products were collected by a permanent magnet and were thoroughly washed with ethanol and deionized water several times. The obtained Fe_3_O_4_ particles were dried at 25 °C under vacuum oven for 24 h.

#### 2.2.2. Synthesis of SA and Fe_3_O_4_-SA-AC Microsphere

A 2.0 g amount of sodium alginate was added to 100 mL distilled water and stirred vigorously for 12 h. A 50 mL aliquot of sodium alginate solution was added slowly into 2% CaCl_2_ solution with a syringe (1 mL). Then, the product (SA) was cured for 30 min, washed several times with deionized water, and sublimated for 12 h. The 50 mL sodium alginate was added into a 250 mL three-neck flask with 0.1 g Fe_3_O_4_. Next, three different amounts of AC (0.1, 0.2 and 0.3 g) were added in the solution, then sonicated and stirred for 1 h. Then, they were stirred an additional 1 h without sonication. The mixed solution was slowly added dropwise into 2% CaCl_2_ solution with a syringe (1 mL). The final products (MSA-AC1, MSA-AC2, and MSA-AC3) were cured for 30 min, collected by a permanent magnet, washed several times with deionized water, and sublimated 12 h.

### 2.3. Batch Experiments

Removal of MB was carried out in a 150 mL Erlenmeyer flask containing MB solution (50 mL) and adsorbent (100 mg). A required amount of experimental parameters influencing the MB dye adsorption, including contact time (0–270 min), initial pH (3–11) of the dye solution, initial concentration (500–700 mg/L), and temperature (298–310 K) were examined. In the adsorption experiments, the pH value of MB solution was maintained by using HCl (0.1 mol/L) or NaOH (0.1 mol/L) solutions. All batch experiments were fully agitated on a BSD-TX345 Thermostat oscillator (Shanghai Instruments Inc., Shanghai, China) at a fixed speed of 150 r/min to attain adsorption equilibrium. Afterward, the adsorbent was separated from the solution by a permanent magnet after adsorption, and the residual concentration of dyes in the solution was analyzed by a UV–Vis spectrophotometer (SHIMADZU, UVmini-1240, Shimadzu Instrument Co., Tokyo, Japan) at the maximum absorbance wavelength of 664 nm (for MB). All samples were measured three times and average values were taken. Adsorption capacity and dye removal efficiency (DRE) were calculated according to Equations (1) and (2):

(1) $$q_{e}=\frac{\left(C_{0}-C_{e}\right)\times V}{W}$$

(2) $$DRE=\frac{\left(C_{0}-C_{e}\right)}{C_{0}}\times 100\%$$

where *q_e_* (mg/g) is adsorption capacity; *C*_0_ (mg/L) and *C_e_* (mg/L) are MB concentrations at initial and equilibrium; *V* (L) is the volume of solution, and *W* (g) is the mass of the adsorbent (dry).

## 3. Results and Discussion

### 3.1. Characterization

#### 3.1.1. FT-IR

**Figure 1:**
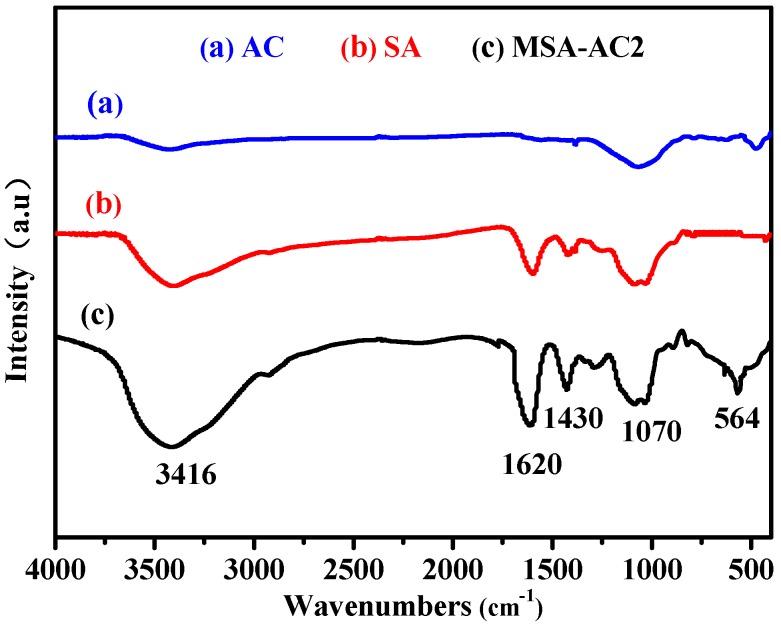
The FT-IR spectra of AC (**a**); SA (**b**); MSA-AC2 (**c**).

FT-IR spectra were measured to further investigate the fabrication process (Figure 1). Characteristic peaks at 1070, 1620 and 3416 cm^−1^ are caused by –OH stretching vibration, 1430 cm^−1^ is attributed to –CH_2_– stretching vibration [20,25]. Compared to the spectra of AC and SA, the new band at 564 cm^−1^ can be attributed to the stretching vibrations of Fe–O in MSA-AC2, and the enhanced peaks 3416 cm^−1^ and 1620 cm^−1^ indicate the presence of Fe_3_O_4_. This result indicated that the MSA-AC composite microspheres successfully cross-linked Fe_3_O_4_ and AC with sodium alginate, and Fe_3_O_4_ had not changed in the cross-linking process.

#### 3.1.2. SEM Analysis

**Figure 2:**
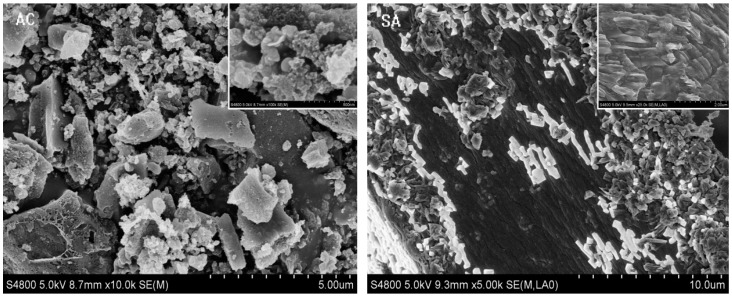

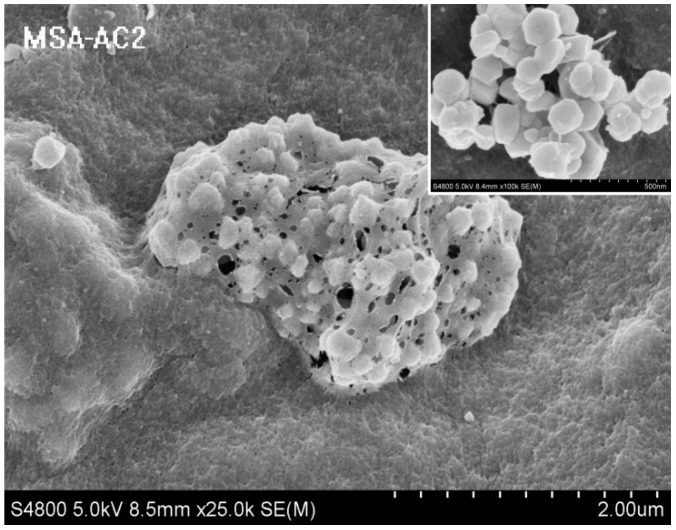
Representative SEM images of SA, AC and MSA-AC2.

The SEM image of the prepared SA, AC and MSA-AC2 materials is shown in Figure 2. The AC displays a fluffy and rough surface with irregular patches and obvious pores. The combination of Fe_3_O_4_ and AC with the cross-linking of sodium alginate (MSA-AC2) has a porous, spongy structure. Additionally, many pores are observed on the outer surface, and the distribution of Fe_3_O_4_ particles were exclusively inset on the surface of hydrogel fiber. This may give rise to some aggregation of Fe_3_O_4_ on the surface, making particles appear larger, as seen in the image [29,30,31].

#### 3.1.3. X-ray Diffraction Analysis

**Figure 3:**
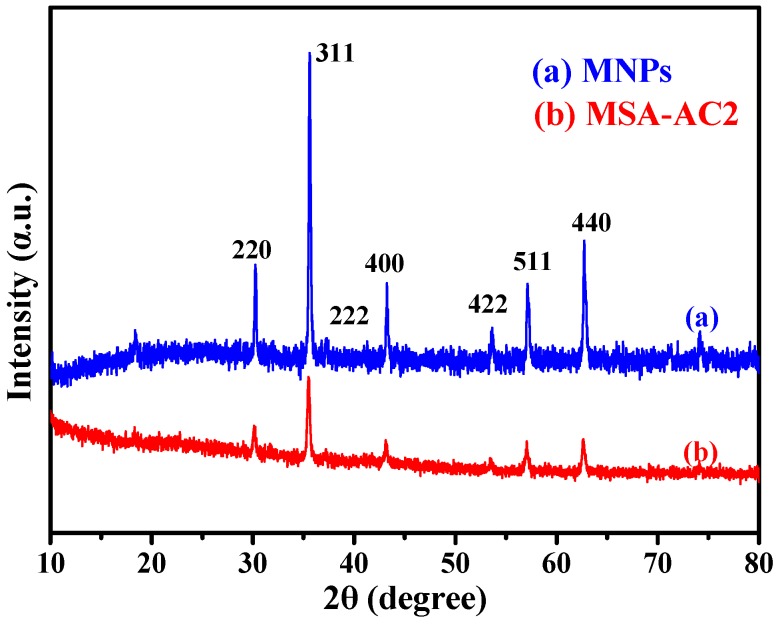
X-ray diffraction patterns of (**a**) MNPs (Fe_3_O_4_ nanoparticles) and (**b**) MSA-AC2.

The X-ray diffraction (XRD) patterns of synthesized Fe_3_O_4_ and MSA-AC2 are displayed in Figure 3. The diffraction peaks of co-preparation Fe_3_O_4_ nanoparticles and Fe_3_O_4_ microspheres occurred at the 2θ region of 20°–90°. The diffraction peaks at 2θ = 30.27°, 35.45°, 37.15°, 43.12°, 53.46°, 57.13°, 62.52° correspond to the (220), (311), (222), (400), (422), (511) and (440) planes, respectively. These diffraction peaks were in accordance with those of the standard magnetic Fe_3_O_4_ XRD pattern (JCPDS, 19-06290). These results indicated that magnetite (Fe_3_O_4_) has a face-centered cubic structure. Moreover, we did not observe other iron oxides, such as α-Fe_2_O_3_, β-Fe_2_O_3_, γ-Fe_2_O_3_, which suggests that Fe_3_O_4_ was not oxidized to other forms in the synthetic steps [24,25,32].

#### 3.1.4. Thermogravimetric Analyzer Analysis

Thermogravimetric analyzer (TGA) measurements were carried out by weighing a powder sample of 5–10 mg and loading it into a platinum pan. The mass change in the temperature range from 30 to 800 °C at a heating rate of 10 °C·min^–1^ under a nitrogen flow was monitored and recorded.

**Figure 4:**
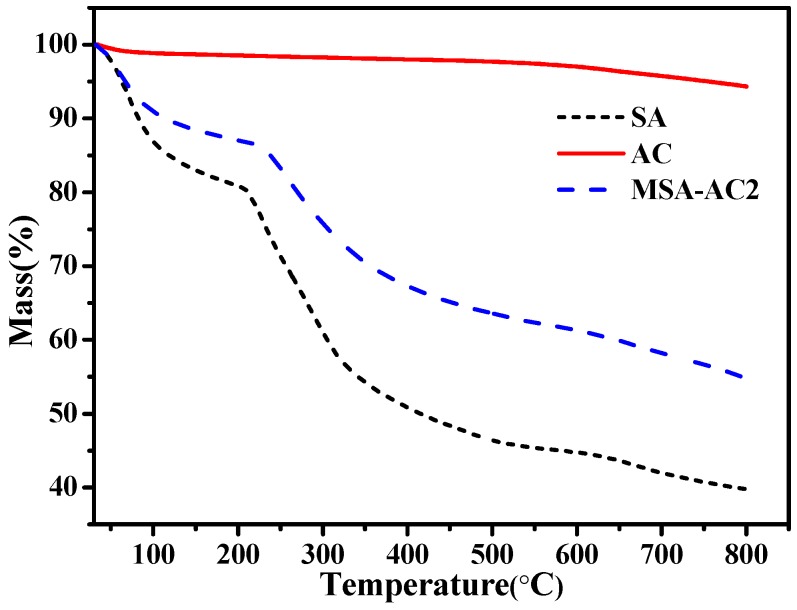
TGA curves of SA, AC and MSA-AC2.

The result of TGA on the microspheres was investigated and presented in Figure 4. The TGA curves of the prepared microspheres gave four mass loss processes before 800 °C. The first mass loss, which was approximately 13.3% at <100 °C, is due to the evaporation of water in the magnetite MSA-AC2. In the second mass loss of about 6.5% between 100 and 220 °C, the decarboxylation of SA released carbon dioxide and part of the products were carbonized. The third mass loss of about 10.7% between 220 and 450 °C is attributed to the thermal decomposition of the aminopropyl groups and bisaldehyde in the nanomaterial. Fourthly, the TGA curve above 600 °C indicates that the organic species in the magnetic composite nanoparticles have been completely decomposed and that the Fe_3_O_4_ nanoparticles remain [33,34].

#### 3.1.5. VSM Analysis

The magnetic characteristics of the particles were measured by a vibration sample magnetometer (VSM) under an applied magnetic field.

**Figure 5:**
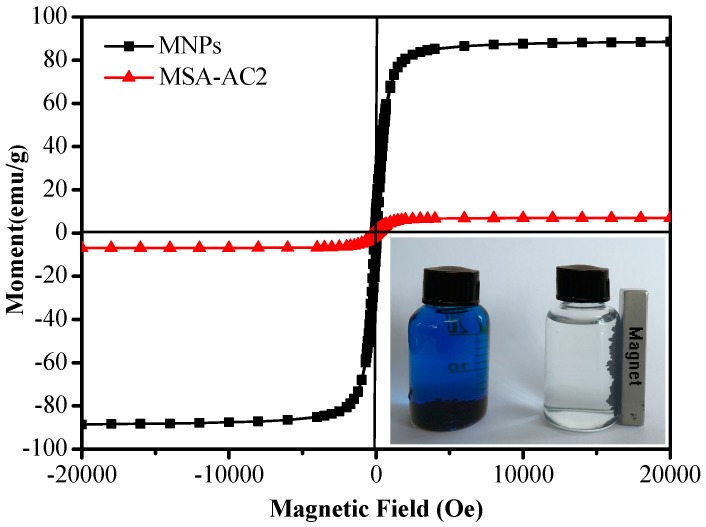
The field-dependent magnetization curve of MNPs (Fe_3_O_4_) and MSA-AC2 at room temperature and a photo of magnetic separation.

The saturation magnetization (Ms) of Fe_3_O_4_ nanoparticles (MNPs) and Fe_3_O_4_-SA-AC (MSA-AC) microspheres was taken at 298 K as shown in Figure 5. The VSM curves of MNPs and MSA-AC resemble those of typical superparamagnetic behavior. Both curves present very narrow hysteresis loops and no remnant magnetization could be observed. The maximum saturation magnetization value of MSA-AC2 (9.01 emu/g) is lower than that for bulk magnetite particles (93 emu/g); however, the MSA-AC2 still has adequate magnetization to be easily and quickly separated from the complex sample. The maximum saturation magnetization value of MSA-AC2 decreases due to the magnetically reducing AC combination; and the assembly of the Fe_3_O_4_ with AC composite microspheres, using sodium alginate as the cross-linker, was beneficial for retaining high saturation magnetization [26,28]. This result might be explained by the unique structure of the MSA-AC2.

#### 3.1.6. BET Analysis

**Figure 6:**
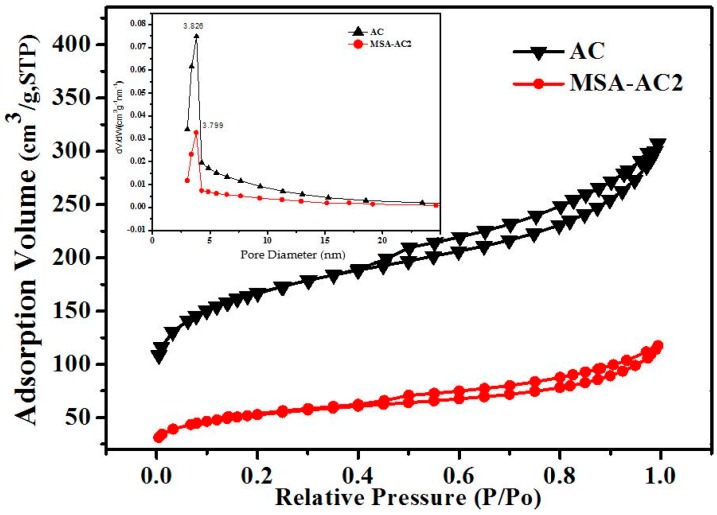
Nitrogen adsorption/desorption isotherms and pore size distribution curves of AC and MSA-AC2.

The N_2_ adsorption/desorption isotherms and pore diameter distribution curves of AC and MSA-AC are shown in Figure 6, and the corresponding parameters of porous structures are listed in Table 1. The pore size distribution suggests that the pore distributions of AC and MSA-AC are 3.826 nm and 3.799 nm, respectively, which is similar to the peak horizontal position (approximately 3.80 nm). Conversely, the pore channels of MSA-AC are more uniform. The surface area and total pore volume are 590.70 m^2^/g, 60.03 m^2^/g, 183.63 m^2^/g, 179.37 m^2^/g and 0.424 cm^3^/g, 0.054 cm^3^/g, 0.153 cm^3^/g, 0.145 cm^3^/g for AC, MSA-AC1, MSA-AC2 and MSA-AC3 respectively. Although the N_2_ adsorption amount of MSA-AC is significantly less than AC, the pore size distribution is basically the same. The N_2_ adsorption/desorption isotherms of AC and MSA-AC2 correspond to a similar Type IV isotherm with Type H2 hysteresis loop behavior (IUPAC, 1984) [35,36], which indicates that (1) the pores of AC and MSA-AC are with predominantly mesopores; (2) capillary condensation will occur in the pores; and (3) compared with AC, the micropore area of MSA-AC had decreased. Thus, MSA-AC has lower surface area and pore volume when compared with AC.

### 3.2. Adsorption Studies

#### 3.2.1. Adsorption Efficiency of Different MNPs with Different Time on Adsorption of MB

**Figure 7:**
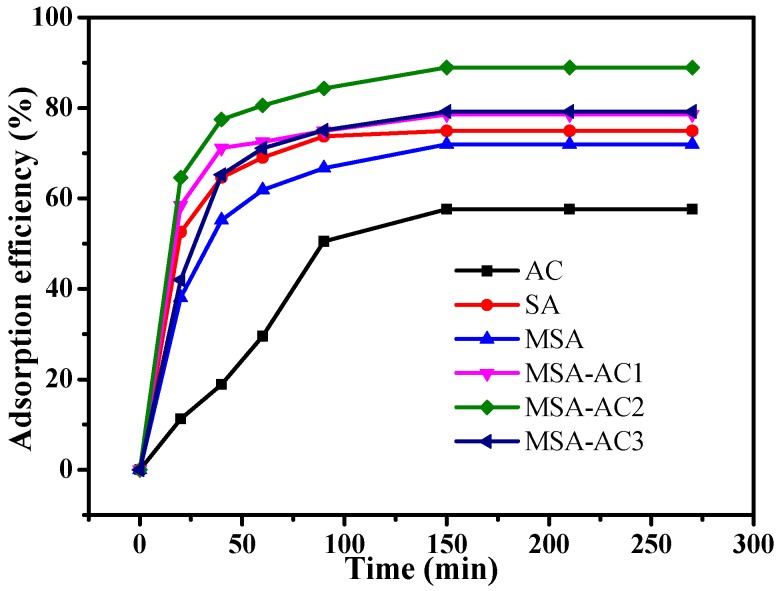
The adsorption capacities of different magnetic spheres over time.

The adsorption efficiencies of AC, SA, MSA, MSA-AC1, MSA-AC2, and MSA-AC3 for MB were evaluated in Figure 7. The adsorption process is divided into two parts. First, MB was quickly adsorbed over 40 min, and the adsorption efficiency of MSA-AC2 achieved was 80%. In the subsequent steps, removal of 89.5% MB was achieved in 150 min. The adsorption percentage was basically unchanged after 150 min. The results were as follows: (1) AC < MSA < SA < MSA-AC1 < MSA-AC3 < MSA-AC2 under the same experimental conditions; (2) adsorption increases with time and an equilibration time of 150 min. Therefore, MSA-AC2 was employed in subsequent experiments. This increase in adsorption is attributed to the synergistic effect of SA and AC, and increase of surface area [15].

#### 3.2.2. Effect of pH on Adsorption of MB

**Figure 8:**
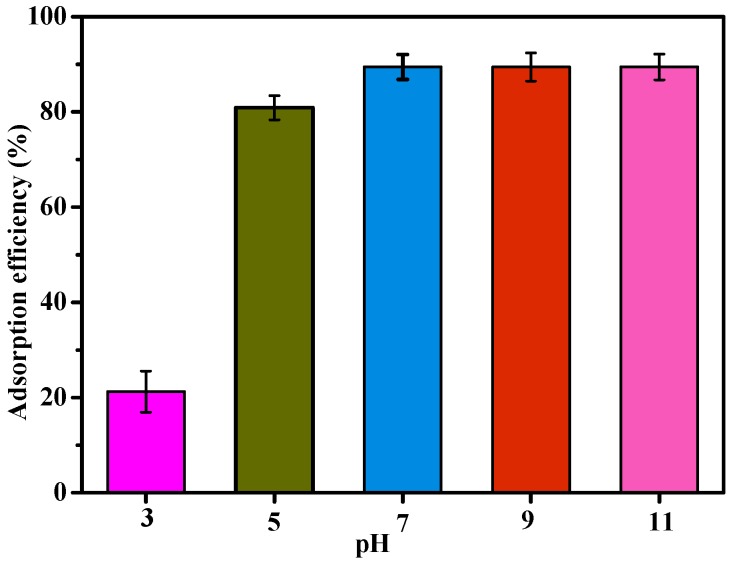
The adsorption efficiency of MSA-AC2 at different pH values.

The pH condition is an important factor during the adsorption process. According to the previous studies [20,31], adsorption of MB was investigated over a pH range from 3 to 11. While pH values were varied, all other conditions were constant: the optimal temperature was 298 K, the initial concentration was 500 mg/L, the optimal time was 150 min, the volume was 50 mL and the adsorbent dose was 100 mg. The adsorption experiments were carried out in triplicate. As shown in Figure 8, the adsorption efficiency varied for pH range 3–7, while efficiency was unaffected for pH range 7–11. At pH 3, the efficiency of MB was only 21.2%. This is likely due to a large number of hydrogen ions in solution that impart a positive charge on the MSA-AC2 microsphere surface, making attachment of MB to the active sites of MSA-AC2 unfavorable [31,37]. The adsorption efficiency of MB increased from 21.2% to 89.5% with increasing pH from 3 to 7, and this can be attributed to protonation of the hydroxyl and carboxyl groups, allowing the formation of a negative surface charge. In short, the adsorption capability was increased at higher pH values because of the stronger electrostatic interaction that existed between MSA-AC2 active sites and MB. This suggested that the optimal adsorption condition of MB, a cationic dye, via MSA-AC2, was under neutral or alkaline pH [38]. Ultimately, pH 7 that was selected as the absorption via MSA-AC2 was highest at 89.5%. 

#### 3.2.3. Effect of Initial Concentration on Adsorption of MB

**Figure 9:**
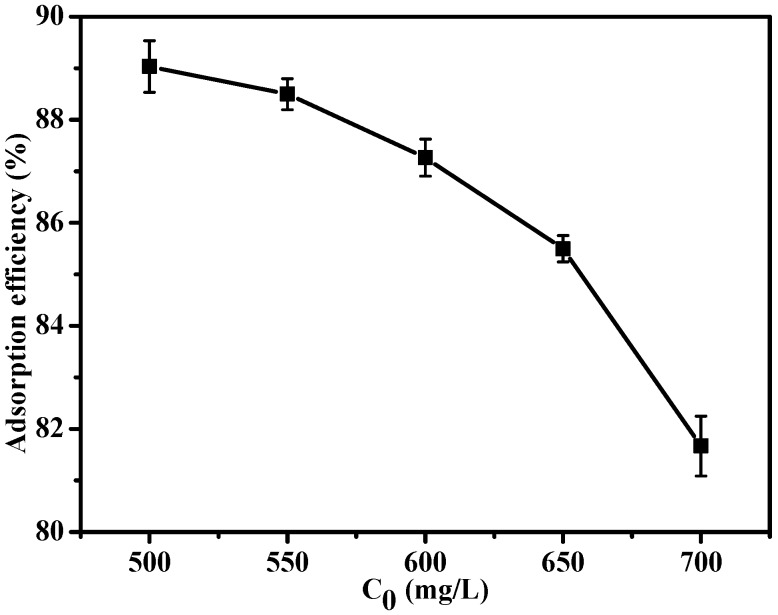
The adsorption efficiency of MSA-AC2 at different initial MB concentrations.

The adsorption efficiency of the MSA-AC2 is shown in Figure 9. The adsorption process reached equilibrium after approximately 150 min (Figure 7), and 298 K was the optimal temperature for all MB solutions. The adsorption efficiency of MSA-AC2 was about 89.0%, 88.5%, 87.3%, 85.5%, and 81.7% at concentrations of 500, 550, 600, 650 and 700 mg/L, respectively. The results indicate that, for MSA-AC2, the adsorption efficiency of MB decreased with increasing MB concentration.

### 3.3. Adsorption Kinetics Studies

To evaluate the adsorption behavior of MB onto MSA-AC2, a pseudo-first-order (Equation (3)) and pseudo-second-order model (Equation (4)) have been applied to analyze the experimental data [39].

(3) $$\frac{1}{q_{t}}=\frac{1}{q_{e}}+\frac{k_{1}}{q_{e}t}$$

(4) $$\frac{t}{q_{t}}=\frac{1}{k_{2}q_{e}^{2}}+\frac{t}{q_{e}}$$

Here, *q_e_* (mg/g) is the equilibrium adsorption capacity, *q_t_* (mg/g) is the adsorption capacity at time t (min), *k*_1_ (1/min) is the pseudo-first-order rate adsorption constant, and *k*_2_ (g/mg/min) is the rate constant of the pseudo-second-order rate adsorption constant. 

**Figure 10:**
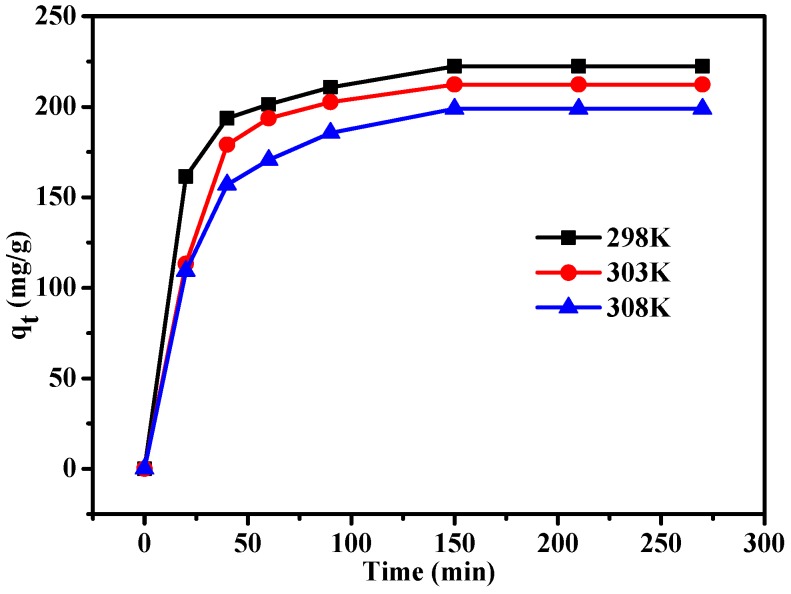
Adsorption kinetics of MSA-AC2.

The kinetic rate constants *k*_1_ and *k*_2_, and the correlation coefficients are listed in Table 2. Figure 10 shows that adsorption increased quickly over 60 min, then reached a saturation point. As temperature increases, adsorption quantity reduced, indicating that low temperature is advantageous to the MSA-AC2 adsorption of MB. According to the correlation coefficient, the experimental data was fit better to the pseudo-second-order model (*R*^2^ ≥ 0.997) than the pseudo-first-order model (*R*^2^ ≥ 0.992). Moreover, the *q_e_* values were obtained for the pseudo-first-order and pseudo-second-order models, and in the case of the pseudo-second-order model, the theoretical value (*q_e,cal_* = 229.89 mg/g, 224.22 mg/g, 211.86 mg/g) was very close to the experimental data (*q_e_* = 222.3 mg/g, 212.3 mg/g, 198.8 mg/g). Therefore, the pseudo-second-order model was used to describe the adsorption of MB on MSA-AC2 [12,39]. In previous studies [31,40], adsorption capacity can be associated with the surface chemical property of MSA-AC.

### 3.4. Adsorption Isothermal Model

Adsorption isotherms are critical for optimizing the use of adsorbents, as they describe how dye molecules interact with adsorbent particles. Therefore, finding the best fitting isotherm is of great importance. The parameters, obtained by fitting the experimental data to Langmuir (Equation (5)) and Freundlich (Equation (6)) isotherms [31] are listed in Table 3.

(5) $$\frac{C_{e}}{q_{e}}=\frac{1}{q_{m}}C_{e}+\frac{1}{q_{m}K_{L}}$$

(6) $$\ln q_{e}=\frac{1}{n}\ln C_{e}+\ln K_{F}$$

Here, *q_e_* (mg/g) is the amount of MB adsorbed at equilibrium, *C_e_* (mg/L) is the equilibrium concentration of MB solution, and *q_m_* (mg/g) is the monolayer adsorption capacity of the adsorbent. *K_L_* is the Langmuir adsorption constant, which is related to the free energy of adsorption. *K_F_* and n (dimensionless) are the Freundlich adsorption isotherm constants, representing the adsorption extent and the degree of non-linearity between solution concentration and adsorption, respectively.

**Figure 11:**
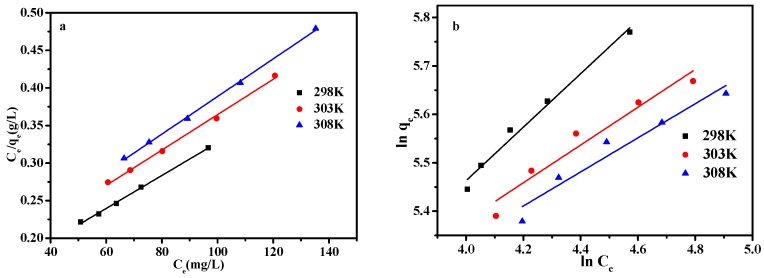
Adsorption isotherm of MB. (**a**) Langmuir isotherm and (**b**) Freundlich isotherm.

**Figure 12:**
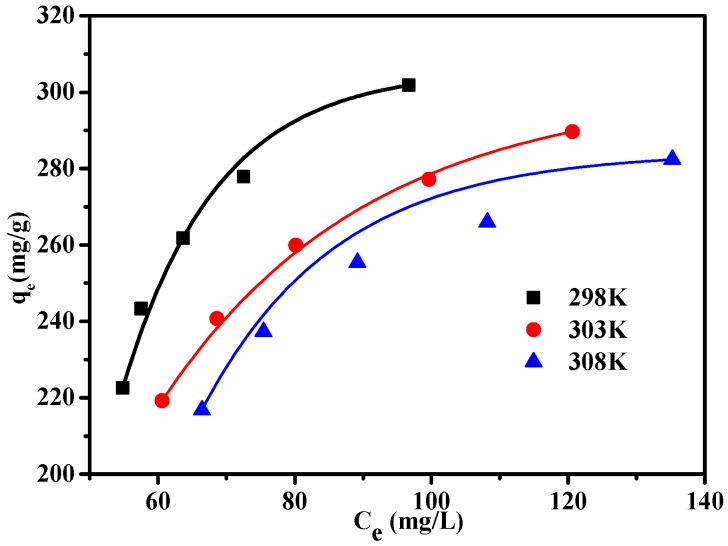
Adsorption isotherms of MSA-AC2 at three temperatures.

The regression parameters calculated from the Langmuir and Freundlich models at three different temperatures (Figure 11) are listed in Table 3. In Figure 12, under the different temperature, the equilibrium adsorption quantity is increased with the increase of the MB solution equilibrium concentration, showing a trend of increasing gradually. The relatively higher *R*^2^ values of Langmuir isotherms, compared with that of Freundlich isotherms, confirm that the Langmuir model (*R*^2^ ≥ 0.996) is a better-fitting isotherm for the experimental data of MSA-AC2. The Langmuir adsorption isotherm equation can better describe the MSA-AC2 adsorption behavior of MB, implying that MB in MSA-AC2 adsorption was monolayer adsorption [31,41]. 

### 3.5. Comparison with Other Methods

When compared with most reported procedures for the determination of MB adsorption, the method described herein is effective for the removal of MB. These results are summarized in Table 4.

## 4. Conclusions

In conclusion, magnetic microspheres were synthesized by a simple and environmentally conscious method. The microspheres effectively adsorbed MB dye and were characterized by FT-IR, SEM, VSM, TGA. These characterization techniques have proved that the Fe_3_O_4_ of MSA-AC2 did not change through the composite process. Although the saturation magnetization of MSA-AC2 was lower than the MNPs, the magnetization of MSA-AC2 was adequate for fast separation. The results demonstrated that MSA-AC2 has high adsorption efficiency, with a maximum adsorption capacity of 222.3 mg/g for MB. The adsorption efficiency for MB was studied under the following conditions: 150 min, 298 K and optimal pH of 7. The as-prepared MSA-AC2 composite exhibited a high BET-specific surface area, reaching 183.63 m^2^/g. The adsorption isotherm fitted well with the Langmuir model. Kinetic data was described appropriately by the pseudo-second-order model (*R*^2^ ≥ 0.999) and the experimental data was very close to the theoretical value of the pseudo-second-order model. These results reveal that the MSA-AC2 has a potential application in wastewater treatment and in the development of a Fe_3_O_4_-SA-AC composite absorbent that is simple and fast to prepare, cost-effective, and environmentally friendly.

## Figures and Tables

**Table 1:** Porous properties of AC and MSA-AC.

Materials	*S_BET_* (m^2^/g) ^a^	Pore Size (nm) ^b^	*S_mic_* (m^2^/g) ^c^	*V_t_* (cm^3^/g) ^d^	*V_mic_* (cm^3^/g) ^e^
AC	590.70	4.93	325.91	0.424	0.142
MSA-AC1	60.03	6.52	33.71	0.054	0.015
MSA-AC2	183.63	5.49	83.63	0.153	0.037
MSA-AC3	179.37	5.58	70.32	0.145	0.031

^a^ BET surface area; ^b^ Barrett, Joyner and Halenda (BJH) model, desorption data; ^c^ Micropore area; ^d^ Total pore volume P/P_o_ = 0.95; ^e^ Micropore volume P/P_o_ = 0.95.

**Table 2:** Kinetic parameters for the adsorption of MB on MSA-AC2.

*T* (K)	Pseudo-First-Order			Pseudo-Second-Order			Experimental Value
	*k*_1_	*q_e,cal_* (mg/g)	*R*^2^	*k*_2_	*q_e,cal_* (mg/g)	*R*^2^	*q_e_* (mg/g)
298	8.63	232.02	0.9925	0.00056	229.89	0.9997	222.3
303	21.73	247.52	0.99209	0.00037	224.22	0.9978	212.3
308	20.10	224.22	0.9933	0.00033	211.86	0.9988	198.8

**Table 3:** Isotherm constants for the adsorption of MB on MSA-AC2.

*T* (K)	Langmuir			Freundlich		
	*K_L_*	*q_m_* (mg/g)	*R*^2^	*K_F_*	1/*n*	*R*^2^
298	0.020	465.12	0.997	25.89	0.552	0.980
303	0.019	418.41	0.996	45.60	0.390	0.930
308	0.021	386.10	0.998	51.06	0.352	0.926

**Table 4:** Adsorbent performance of MB in literature.

Adsorbent	*q_e_* (mg/g)	Reference
Fe_3_O_4_/SA/AC	222.3	Our Work
SiO_2_@poly(SVS-co-ITA–DA)	111.4	[42]
Mesoporous Fe_3_O_4_@SiO_2_	33.1	[43]
Magnetic Activated Carbons	871	[44]

## References

[1] Sirianuntapiboon S., Srisornsak P. (2007). Removal of disperse dyes from textile wastewater using bio-sludge. Bioresour. Technol..

[2] Ali I., Gupta V.K. (2006). Advances in water treatment by adsorption technology. Nat. Protoc..

[3] Cai T., Yang Z., Li H., Yang H., Li A., Cheng R. (2013). Effect of hydrolysis degree of hydrolyzed polyacrylamide grafted carboxymethyl cellulose on dye removal efficiency. Cellulose.

[4] Han X., Dong S.Y., Yu C.F., Wang Y.Y., Yang K., Sun J.H. (2016). Controllable synthesis of Sn-doped BiOCl for efficient photocatalytic degradation of mixed-dye wastewater under natural sunlight irradiation. J. Alloys Compd..

[5] Homaeigohar S., Zillohu A.U., Abdelaziz R., Hedayati M.K., Elbahri M. (2016). A Novel Nanohybrid nanofibrous adsorbent for water purification from dye pollutants. Materials.

[6] Zhu Y.F., Zheng Y.A., Wang F., Wang A.Q. (2016). Monolithic supermacroporous hydrogel prepared from high internal phase emulsions (HIPEs) for fast removal of Cu^2+^ and Pb^2+^. Chem. Eng. J..

[7] Cheng C., Deng J., Lei B., He A., Zhang X., Ma L., Li S.A., Zhao C.S. (2013). Toward 3D graphene oxide gels based adsorbents for high-efficient water treatment via the promotion of biopolymers. J. Hazard. Mater..

[8] Cui W., Ji J., Cai Y.F., Li H., Ran R. (2015). Robust, anti-fatigue, and self-healing graphene oxide/hydrophobic association composite hydrogels and their use as recyclable adsorbents for dye wastewater treatment. J. Mater. Chem. A.

[9] Chew C.M., Aroua M.K., Hussain M.A., Ismail W.M.Z.W. (2015). Practical performance analysis of an industrial-scale ultrafiltration membrane water treatment plant. J. Taiwan Inst. Chem. E.

[10] Laia G.S., Laua W.J., Grayb S.R., Matsuuraa T., Jamshidi Goharia C.R., Subramaniana M.N., Laid S.O., Onge C.S., Ismaila A.F., Emazadaha D. (2016). A practical approach to synthesize polyamide thin film nanocomposite (TFN) membrane with improved separation properties for water/wastewater treatment. J. Mater. Chem. A.

[11] Le C.C., Kunacheva C.G., Stuckey D.C. (2016). “Protein” measurement in biological wastewater treatment systems: A critical evaluation. Environ. Sci. Technol..

[12] Suárez L., Pulgarin C., Roussel C., Kiwi J. (2016). Preparation, kinetics, mechanism and properties of semi-transparent photocatalytic stable films active in dye degradation. Appl. Catal. A Gen..

[13] Rafatullah M., Sulaiman O., Hashim R., Ahmad A. (2010). Adsorption of methylene blue on low-cost adsorbents: A review. J. Hazard. Mater..

[14] Valix M., Cheung W.H., McKay G. (2006). Roles of the textural and surface chemical properties of actived carbon in the adsorption of acid blue dye. Langmuir.

[15] Hu S., Hsieh Y.L. (2014). Preparation of activated carbon and silica particles from rice straw. ACS Sustain. J. Chem. Eng..

[16] Stone M.T., Kozlov M. (2014). Separating proteins with activated carbon. Langmuir.

[17] Hadi P., Guo J.X., Barford J., Mckay G. (2016). Multilayer dye adsorption in activated carbons-facile approach to exploit vacant sites and interlayer charge interaction. Environ. Sci. Technol..

[18] Huang Y.X., Ma E., Zhao G.J. (2015). Preparation of liquefied wood-based activated carbon fibers by different activation methods for methylene blue adsorption. RSC Adv..

[19] He Y.Q., Zhang N.N., Gong Q.J., Qiu H.X., Wang W., Liu Y., Gao J.P. (2012). Alginate/graphene oxide fibers with enhanced mechanical strength prepared by wet spinning. Carbohydr. Polym..

[20] Rocher V., Bee A., Siaugue J.M., Cabuil V. (2010). Dye removal from aqueous solution by magnetic alginate beads crosslinked with epichlorohydrin. J. Hazard. Mater..

[21] Bunkoed O., Kanatharana P. (2015). Extraction of polycyclic aromatic hydrocarbons with a magnetic sorbent composed of alginate, magnetite nanoparticles and multiwalled carbon nanotubes. Microchim. Acta.

[22] Giakisikli G., Anthemidis A.N. (2013). Magnetic materials as sorbents for metal/metalloid preconcentration and/or separation. A review. Anal. Chim. Acta.

[23] Wang W.B., Kang Y.R., Wang A.Q. (2013). One-step in situ fabrication of a granular semi-IPN hydrogel based on chitosan and gelatin for fast and efficient adsorption of Cu^2+^ ion. Colloid Surf. B.

[24] Shen J.H., Zhu Y.H., Yang X.L., Zong J., Li C.Z. (2013). Multifunctional Fe_3_O_4_@Ag/SiO_2_/Au core-shell microspheres as a novel SERS-activity label via long-range plasmon coupling. Langmuir.

[25] Villa S., Caratto V., Locardi F., Alberti S., Sturini M., Speltini A., Maraschi F., Canepa F., Ferretti M. (2016). Enhancement of TiO_2_ NPs activity by Fe_3_O_4_ nano-seeds for removal of organic pollutants in water. Materials.

[26] Yang Q., Lan F., Yi Q.Y., Wu Y., Gu Z.W. (2015). A colloidal assembly approach to synthesize magnetic porous composite nanoclusters for efficient protein adsorption. Nanoscale.

[27] Wan H., Huang J.F., Liu Z.S., Li J.A., Zhang W.B., Zou H.F. (2015). Dendrimer-assisted magnetic graphene—Silica hydrophilic composite for efficient and selective enrichment of glycopeptides from the complex sample. Chem. Commun..

[28] Chen H.M., Deng C.H., Zhang X.M. (2010). Synthesis of Fe_3_O_4_@SiO_2_@PMMA core-shell-shell magnetic microspherss for highly efficient enrichment of peptides and proteins for MALDI-ToF MS analysis. Angew. Chem. Int. Ed..

[29] Kim K.D., Kim S.S., Choa Y.H., Kim H.T. (2007). Formation and surface modification of Fe_3_O_4_ nanoparticles by co-precipitation and sol-gel method. Ind. Eng. Chem..

[30] Laurent S., Forge D., Port M., Roch A., Robic C., Elst L.V., Muller R.N. (2008). Magnetic Iron oxide nanoparticles: Synthesis, stabilization, vectorization, physicochemical characterizations, and biological applications. Chem. Rev..

[31] Karaer H., Kaya İ. (2016). Synthesis, characterization of magnetic chitosan/active charcoal composite and using at the adsorption of methylene blue and reactive blue4. Microporous Mesoporous Mater..

[32] Sreeja N., Binulal N.S., Ullas M., Manzoor K., Shantikumar V.N., Deepthy M. (2011). Biocompatible magnetite/gold nanohybrid contrast agents via green chemistry for MRI and CT bioimaging. ACS Appl. Mater. Interfaces.

[33] Khairou K.S. (2002). Kinetics and mechanism of the non-isothermal decomposition: I. Some divalent cross-linked metal-alginate ionotropic gels. J. Therm. Anal. Calorim..

[34] Kok M.V. (2007). Non-isothermal dsc and TG/DTG analysis of the combusion of silopiasphaltites. J. Therm. Anal. Calorim..

[35] Wang Y.X., Sun H.Q., Ang H.M., Tade M.O., Wang S.B. (2014). Magnetic Fe_3_O_4_/carbon sphere/cobalt composites for catalytic oxidation of phenol solutions with sulfate radicals. Chem. Eng. J..

[36] Xu Z.J., Lu J.H., Liu Q., Duan L., Xu A.H., Wang Q., Li Y.G. (2015). Decolorization of Acid Orange II dye by peroxymonosulfate activated with magnetic Fe_3_O_4_@C/Co nanocomposites. RSC Adv..

[37] Liu Q.S., Zheng T., Li N., Wang P., Abulikermu G. (2010). Modification of bamboo-based activated carbon using microwave radiation and its effects on the adsorption of methylene blue. Appl. Surf. Sci..

[38] Zhang Y., Wang W.B., Zhang J.P., Liu P., Wang A.Q. (2015). A comparative study about adsorption of natural palygorskite for methylene blue. Chem. Eng. J..

[39] Benhouria A., Islam M.A., Zaghouane-Boudiaf H., Boutahala M., Hameed B.H. (2015). Calcium alginate–bentonite–activated carbon composite beads as highly effective adsorbent for methylene blue. Chem. Eng. J..

[40] Kim C., Zhang Z.F., Wang L.S., Sun T., Hu X.M. (2016). Core–shell magnetic manganese dioxide nanocomposites modified with citric acid for enhanced adsorption of basic dyes. J. Taiwan Chem. E.

[41] Allaboun H., Abu Al-Rub F.A. (2016). Removal of 4-chlorophenol from contaminated water using activated carbon from dried date pits: Equilibrium, kinetics, and thermodynamics analyses. Materials.

[42] Huang Q., Liu L.C., Zeng G.J., Liu M.Y., Mao L.C., Huang H.Y., Deng F.J., Zhang X.Y., Wei Y. (2016). Preparation of silica nanoparticle based polymer composites via mussel inspired chemistry and their enhanced adsorption capability towards methylene blue. RSC Adv..

[43] Tan X.J., Lu L.J., Wang L.Z., Zhang J.L. (2015). Facile synthesis of bimodal mesoporous Fe_3_O_4_@SiO_2_ composite for efficient removal of methylene blue. Eur. J. Inorg. Chem..

[44] Gao S.S., Liu L., Tang Y.K., Jia D.Z., Zhao Z.B., Wang Y.Y. (2016). Coal based magnetic activated carbon as a high performance adsorbent for methylene blue. J. Porous Mater..

